# Epidermal Stem Cells in Orthopaedic Regenerative Medicine

**DOI:** 10.3390/ijms140611626

**Published:** 2013-05-31

**Authors:** Jin Li, Gehua Zhen, Shin-Yi Tsai, Xiaofeng Jia

**Affiliations:** 1 Biomedical Engineering, the Johns Hopkins University School of Medicine, 720 Rutland Ave., Traylor #710B, Baltimore, MD 21205, USA; E-Mails: lijinunion@gmail.com (J.L.); shintsai@jhsph.edu (S.-Y.T.); 2 Orthopaedic Surgery, the Johns Hopkins University School of Medicine, 720 Rutland Ave., Ross #229, Baltimore, MD 21205, USA; E-Mail: gzhen1@jhmi.edu; 3 Anesthesiology and Critical Care Medicine, the Johns Hopkins University School of Medicine, 720 Rutland Ave., Traylor #710B, Baltimore, MD 21205, USA; 4 Physical Medicine and Rehabilitation, the Johns Hopkins University School of Medicine, 720 Rutland Ave., Traylor #710B, Baltimore, MD 21205, USA

**Keywords:** epidermal stem cells, wound healing, peripheral nerve injury, spinal cord injury, molecular biology, orthopedic regenerative medicine

## Abstract

In the last decade, great advances have been made in epidermal stem cell studies at the cellular and molecular level. These studies reported various subpopulations and differentiations existing in the epidermal stem cell. Although controversies and unknown issues remain, epidermal stem cells possess an immune-privileged property in transplantation together with easy accessibility, which is favorable for future clinical application. In this review, we will summarize the biological characteristics of epidermal stem cells, and their potential in orthopedic regenerative medicine. Epidermal stem cells play a critical role via cell replacement, and demonstrate significant translational potential in the treatment of orthopedic injuries and diseases, including treatment for wound healing, peripheral nerve and spinal cord injury, and even muscle and bone remodeling.

## 1. Introduction

Skin epidermis is a stratified epithelium, which acts as a protective barrier against physical or chemical stress and microorganisms. To serve its protective role, the epidermis gains the ability to continuously renew itself and its appendages during its evolutionary process. In adults, epidermis-resident progenitor cells adopt their fates to fit the renewal of the epidermis, hair follicle, and sebaceous gland and maintain a homeostasis in normal tissue. This renewability, or the regenerative capacity indicates the existence of stem/progenitor cells, which could potentially be harnessed in regenerative medicine.

The first primitive cells were successfully isolated from hair follicles in 1990 [1,2]. The undifferentiated cells exhibited self-renewing and multipotent differentiation capacities and gave rise to all component cells of epidermis and hair follicle [1,2]. These cells were considered to be epidermal stem cells (ESCs), and the follicle was believed to be the nest of ESCs. However, further studies illustrated that the sebaceous gland and the interfollicle epidermis (IFE) were also capable of self-renewal themselves without the hair follicle [3–7]. Thus, the follicle-derived stem cells were termed as hair follicle stem cells (HFSCs). In addition, melanocytes, which also reside in the epidermis, originally derive from neural crest. Recently, several pluripotent stem cell populations from the follicle bulge, expressing characteristic neural crest cell markers, were named epidermal-resident neural crest stem cells (EPI-NCSCs) [8–10] or as follicle pluripotent stem cells (fPSCs) [11–14]. fPSCs are more primitive than hair follicle stem cells (HFSCs) and HFSCs were considered to be daughter cells of fPSCs [8,15,16].

## 2. Populations of ESCs

Epidermal stem/progenitor cells are not a homogenous population and are comprised of different subpopulations. These subpopulations are different in their molecular characteristics, their differentiation capacity, and their resident sites. They have been found in almost every part of the epidermis, including the interfollicular epidermis, in the region of the outer root sheath of the hair follicle, in the hair follicle, the sebaceous gland and sweat gland.

### 2.1. HFSCs

HFSCs were the first stem cells isolated from epidermis and the most recognized stem cells. HFSCs are isolated from the bulge area, not from the bulb of the mouse hair follicle. Human HFSCs are also founds in the hair follicle bulge, though the bulge in human is not so clear as in the mouse [17,18]. In adult, HFSCs have the highest colony-forming efficiency compared with other slow-cycle cells from epidermis, and are capable of differentiation into every kind of epidermal cell [1,2]. Normally, HFSCs are only involved in the renewal of the hair follicle [3–7]. HFSCs have the ability to participate in the reconstruction of the hair follicle, the interfollicle epidermis and the sebaceous gland. Thus, HFSCs can be a potential treatment for skin defects, due to their capability of giving rise to epidermal, hair follicle, and sebaceous glands.

### 2.2. Interfollicle Epidermal Stem Cells (IFESCs)

IFESCs are putative stem cells but currently still lack full identification and verification. IFESCs are speculated to sporadically reside in the basal layer of the epidermis. A controversy exists on whether there are IFESCs in the epidermis or not [19]. Some studies strongly supported its presence in the epidermis. For example, new epidermis can regenerate in the absence of HFSCs in acute wound closure [20]. In addition, genetic lineage tracing has revealed that the interfollicle epidermis contains other long-lived multipotent population of stem cells. These cells are responsible for maintaining homeostasis of the interfollicle epidermis [3–7]. However the exact isolation of IFESCs has been unsuccessful except for one case from the mouse tail [19].

### 2.3. Sebaceous Gland Stem Cells (SGSCs)

The SGSCs reside in the base of the sebaceous gland and are able to maintain sebaceous gland homeostasis without recruiting HFSCs [5,21,22]. Genetic lineage tracing has shown that the Blimp1+ SGSCs could give rise to all cells within the SG but not the cells within the hair follicle and epidermis during remodeling [22].

### 2.4. fPSCs

Since identification of nestin+ cells was reported in the hair follicle bulge in 2003, the pluripotent stem cells have generated great interest in the scientific community [23]. These primitive cells express nestin instead of keratin-15 that is a marker for HFSCs [24,25]. They can differentiate into progenies more diverse than the component cells of dermis [8–10], including neuron, glial cells, smooth muscle cells, chondrocytes, and osteocytes. These nestin+ primitive cells and embryonic neural crest cells commonly express a panel of 19 genes, which represent the characteristics of neural crest cells [26,27]. Thus, these nestin+ primitive cells were termed EPI-NCSCs [8–10]. However, nestin, which was previously considered to be a marker for primitive neural cells, is found in many other kinds of adult stem cells, such as mesenchymal stem cells. Thus, these pluripotent stem cells from follicle are termed nestin+ fPSCs as well [28,29].

Two kinds of Lgr6+ pluripotent stem cells were reported. One was isolated from hair follicle with the capacity of differentiating into mesenchymal lineages (adipocytes, chondrocytes, muscle cells) or neurons (βIII-tubulin) [16]. The other was isolated from a small part of the follicle, the central isthmus directly above the bulge. These cells participated in renewal of sebaceous glands and interfollicular epidermis postnatally [14]. However, the relation of these two Lgr6+ primitive cells is unclear.

With the exception of one study that showed nestin+ fPSCs expressed Lgr6 [29], there has been no further evidence to support that nestin+ fPSCs and Lgr6+ fPSCs are the same one. The distinct difference between FPSCs and HFSCs is that FPSCs are keratin15 negative. FPSCs can differentiate into multiple progenies, which distinguish it as a compelling therapeutic modality in clinical application [8–10].

**Figure 1:**
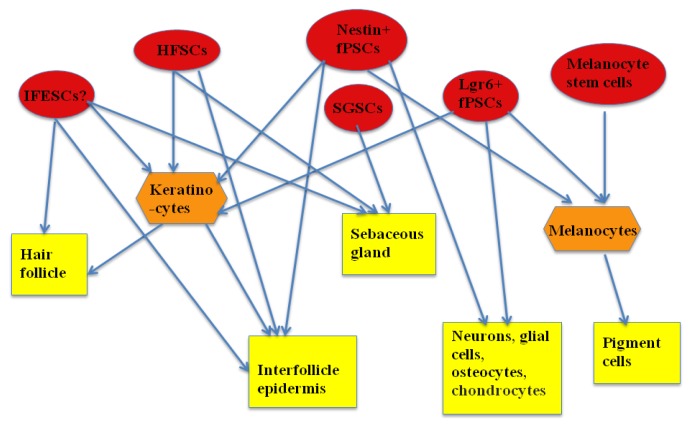
The population of epidermal stem cells (ESCs) and their progenies. The arrow illustrates differentiation from the stem (in red oval)/progenitor cells (in orange hexagonal) to the progeny, or the relation between the stem cells and the tissue, whose renewal the stem cells participate in. IFESCs, interfollicle epidermis stem cells; HFSCs, hair follicle stem cells; fPSCs, follicle pluripotent stem cells; SGSC, sebaceous gland stem cells. “IFESCs?” represents a controversy in IFESCs.

In addition, melanocyte stem cells [30–33] and sweat gland stem cells [34] have been reported. Melanocyte stem cells reside in the hair follicle bulge, cooperate with HFSCs to maintain the stem cell niche, and initiate pigmented hair regeneration. Unipotent melanocyte stem cells differentiate into melanocytes and further into pigment cells. Sweat gland stem cells only give rise to the cells of the sweat gland. It was recently reported that adult myo-epithelial and sweat duct progenitors from sweat ducts have multi-potential and can regenerate sweat glands [34]. The clinical potential of these two unipotent stem cells has not yet been reported. The summary of interaction among the population of ESCs and their progenies can be seen in Figure 1.

Instead of a combination of stem cells from multiple origins existing in the epidermis, some researchers still insist that ESC is a single stem cell with a proliferative hierarchy [35–38]. The disagreement on the phenotype of the epidermal-residential stem cell profoundly influenced their application in regenerative medicine. This controversy will remain until the relationship among these different stem cells is full identified. The current focus of ESC research is to address this controversy includes a novel isolating technique, a new molecular marker, a new component of epidermal-resident stem cells, and elucidation of the relationships among those epidermal-resident stem/progenitor cells.

## 3. Molecular Signal of ESCs

### 3.1. Molecular Signal of HFSCs

Until recently, the understanding of molecular signaling of ESCs mainly comes from the HFSCs. Typically, HFSCs with a special molecular marker known as cytokeratin15 [17] maintain a balance between quiescence and proliferation or asymmetric differentiation [39]. The expression of integrin β1 was reported in several lineages of adult and embryonic stem cells, which suggested it as a sign of “stemness” [40,41]. A log linear relationship between the relative expressive level of integrin β1 on the cell surface and proliferative capacity of keratinocyte stem cells was reported in 1993, but it was still difficult to understand the exact role of integrin β1 on keratinocyte stem cells [42,43].

Wnt/β-catenin is a crucial signal in the hair follicle. Wnt signal, via induction of Tcf3 transcription factor, maintains a multipotent cell phenotype of HFSCs [44–47]. In addition, the Wnt signal could regulate the epidermal lineage selection during differentiation [48]. As a downstream gene of Wnt/β-catenin signal, *Myc* triggers an ESC proliferation by regulating some genes including *integrin β1*, *integrin α6*, and *Misu*, a RNA methyltransferase [49,50]. Both *Rac1* (by downregulating c-Myc through p21 activated kinase 2 (PAK2) phosphorylation) and *Ovol1* (repressing c-myc transcription by directly binding to its promoter) expression suppress proliferation and maintain the quiescence of ESCs [51,52].

*Sonic hedgehog* (*Shh*) is another key gene in hair development and hair cycle maintenance postnatally [53]. *Shh* mutation leads to large and abnormal follicles with a high proliferation of cells and hair-shaft-like material without hair [54]. Application of anti-hedgehog monoclonal antibodies can block hair development completely, and inhibit the normal hair cycles in young and adult mice [55].

*Notch ligand Delta 1* is expressed in the basal layer of human epidermis, with particularly high expression where HFSCs reside, and have a putative role in maintaining HFSC quiescence [56]. Notch signal accelerates keratinocytes differentiation after HFSCs exit the stem cell niche [57].

Other signaling molecules, including *epidermal growth factor receptor* (*EFGR*) [58,59] and *P63* [56,60,61], are involved in hair cycle and epidermal renewal. All these signal molecules cooperate with the matrix and constitute a complex signaling network—the ESCs niche.

### 3.2. Molecular Biology in Other Components

For SGSCs, Blimp1 is not only a molecular marker, but also acts as a gatekeeper to the SG progenitor cell [22]. Blocking of Blimp1 signal, results in increased sebacyte proliferation and expansion of the glands. Blimp-1 signal regulates sebaceous gland cell proliferation via *c-Myc* gene. Shh signal and Wnt signal are the major players in the sebacyte proliferation [62–64]. Yet, very little is currently known about the molecular biology of IFESCs and fPSCs.

Understanding how those stem cells maintain homeostasis between quiescence and symmetric differentiation or activation in the wound is a major challenge in ESCs biology. New investigations continuously proceed to unveil the molecular biology in the epidermal renewal and would healing process, and will demonstrate further implication on the application of epidermal-resident stem cell on regenerative medicine.

## 4. Application of ESCs in Orthopedics Regenerative Medicine

### 4.1. Advantages of ESCs in Regenerative Medicine

ESCs represent a promising candidate for regenerative medicine because of the following advantages: (1) Immune-privilege and immunomodulatory in transplantation as allogeneic cells; (2) A high degree of inherent plasticity, which assists in the adoption of ESCs by the recipient tissue and allows for the differentiation into desirable progenies similar to the recipient tissue; (3) A large volume of stem cells is easy to obtain; (4) The well-known and easily accessible locations from the human body to get ESCs; (5) No known risk of oncogenesis.

### 4.2. Risk of Oncogenesis of ESCs after Transplantation

Stem cell transplantation, autologous or allogeneic, imposes a potential risk of oncogenesis, especially with some induced stem cells. However, no tumor was found in an animal study transplanted with fPSCs after a six-month follow-up [65], although more research and longer term observations are needed to fully address the issue.

### 4.3. Preparation of ESCs for Regenerative Medicine

It has been found that the niche is important for ESCs to maintain the homeostasis between quiescence and symmetric differentiation. The stem cell niche cannot be duplicated *ex vivo* because the complexity of its ultra-circumstance has not been fully elucidated. To obtain a large amount of primitive ESCs in a short term is important for stem-cell therapy.

#### 4.3.1. Preparation of HFSCs

HFSCs are prone to differentiate in the conventional two-dimensional cell culture. New studies attempt to utilize a three-dimensional (3D) culture model or basement membrane but current protocols are not well-accepted for expansion of HFSCs *in vitro*. For example, in a 3D culture model [66], mitomycin-*C*-inhibited 3T3-J2 fibroblasts were cultured in rat-tail-collagen-I coated dishes as feeder cells. HFSCs were cultured for the first 8 passages with these feeder cells. Then HFSCs were able to keep their stemness in the next expansion to 20 passages without the feeder cells [66]. Clonal growth analysis showed approximately 29% holoclones, 54% meroclones and 17% paraclones. A high ratio of cells kept in primitive state with expression of *keratin 15*, *Sox9*, *NFATc1* and *Zfp145*. These HFSCs could transform into epidermal equivalents when cultured at the air-liquid interphase in high calcium medium [66]. A large number of HFSCs could be derived with this effective cultural method, but some of them started differentiation.

In another study, human HFSCs were cultured in Matrigel basement membrane matrix (BD Biosciences, San Jose, CA, USA) to avoid significant changes in morphology and molecular phenotypes. After 4 weeks, the number of these cells could be expanded two-fold and after magnetic separation the percentage of CD200+ primitive hHFSCs was found up to 78.2% [67]. Although this is not a large-scale expansion, this method is good to keep HFSCs in an undifferentiating state.

Ji *et al.* tried to mimic the stem cell niche with an amniotic membrane. A 3D micronized (300–600 mm) amniotic membrane (mAM) was prepared by repeated freeze-thawing in liquid nitrogen. The mAM retained the basement membrane structure and held trophins such as NGF, HGF, bFGF, TGF-b1 and EGF. When cultured in the mAM in the rotary cell culture system, HFSCs expanded quickly and maintained the characteristics of stem cells with reduced differentiation. The relative proliferative rate of HFSCs in mAM reached 326% ± 28% and 535% ± 47% by day 3 and 14 respectively, *versus* 232% ± 21%, 307% ± 32% in the conventional plate culture (*p* < 0.05). The most prominent advantage was that the mAM with ESCs gradually turn into a new epidermis, when transplanted to full-thickness skin defects in nude mice [68].

#### 4.3.2. Preparation for fPSCs

There are several kinds of fPSCs. Their culture and expansion methods are different. It always takes more than six weeks to get enough amounts of fPSCs [12,13,69]. There is no method to expand these fPSCs to a large-scale while still maintaining the stemness in a short-term. Here, we introduce a well-accepted protocol to prepare the nestin+ fPSCs.

Under a microscope, whisker follicles were dissected and the bulge region was obtained. The bulges were placed into collagen-coated culture plates and cultured in a medium containing 85% Alpha-modified MEM, 10% fetal calf serum and 5% day-11 chick embryo extract. Four days later, adherent cells were transferred and re-suspended in expansion medium. The expansion medium consisted of 90% alpha- modified MEM, 10% day-11 chick embryo extract, 5 ng/mL murine stem cell factor, 20 ng/mL fibroblast growth factor-2, and 20 ng/mL epidermal growth factor [70]. Even with this optimized culture medium, it still took 6–8 weeks to reach enough amounts of nestin+ fPSCs for experimental application.

## 5. ESCs in Skin Regeneration and Wound Healing

### 5.1. ESCs Biology in Skin Regeneration and Wound Healing

Physiologically, HFSCs in the intact epidermis are only involved in the cell-replacement of hair follicles during the hair cycle; IFESCs [3] are responsible for maintaining the interfollicle epidermal lineages, and the SGSCs [22] for the whole gland lineages. In some conditions, HFSCs and IFESCs can differentiate into all the components of the cutaneous epithelium: epidermis, hair follicles and sebaceous glands [2]; SGSCs can differentiate into the component cells of the sebaceous gland postnatally [22]. It has been reported that a wide variety of epidermis-resident stem cells were involved in the process of wound healing [43].

After skin injury, the HFSCs are recruited into the epidermis and migrate in a linear manner toward the center of the wound. They subsequently differentiate into interfollicular keratino–cytes and contribute ~30% of cells to the newly formed epidermis [3]. Integrin-linked kinase (ILK) was a key factor for bulge stem cell differentiation to the interfollicular epidermis after injury. ILK-deficient HFSCs produced a few progenies that moved toward the epidermal surface and into the advancing epithelium. Furthermore, ILK-deficient-HFSCs mice had a significantly reduced number of interfollicle keratinocytes and spent less resident time in the new dermis, which impaired cutaneous wound healing with substantially decreased wound closure rates [71]. Without the hair follicles and HFSCs, the wound can be healed by recruiting IFESCs from a larger area of interfollicular epidermis with an extension of time [20].

In addition, nestin+ fPSCs were reported to participate in skin remodeling [8]. In wounded skin, clusters of nestin+ fPSCs were detected outside the bulge and these cells were found to move toward the epidermis and participate in the wound-healing processes [15].

Understanding the biology of ESCs in skin regeneration and wound healing can facilitate clinical wound treatment in clinic. For example, Ent-16a, 17-dihydroxy-kauran-19-oic acid (DHK) promoted proliferation and migration of ESCs via the epithelial growth factor receptor (EGFR)-Akt/ERK pathway [72]. In animal wound model created by laser burn, DHK significantly accelerated wound healing and increased re-epithelialization by day 4 and 9, which suggest DHK might be applied in the wound-healing process [72].

### 5.2. ESCs in Skin Tissue Engineering for Wound Healing

Clinically, a large-area skin defect, such as those secondary to burn or skin avulsions, necessitates skin graft or skin substitution. ESCs are an important component in the generation of skin-like structures for transplantation in skin tissue engineering. Now there are many kinds of artificial skins applied in clinic. Skin substitution can be categorized into three kinds, (1) grafts consisting of epidermal component alone, (2) grafts consisting of dermal component alone, and (3) grafts containing both dermal component and epidermal components.

Skin substitutions, consisting of epidermal component alone, always have an issue to maintain the thickness of skin after transplantation. The dermal fibroblasts and other dermal components are able to improve the proliferation and differentiation of keratinocytes, and maintain the normal epidermis [73]. A ‘sandwich’ organotypic culture consisting of postmitotic fibroblasts (acting as feeder cells), artificial basement membrane and outer root sheath cells (largely comprised of undifferentiated keratinocytes), could differentiate into epidermal equivalents [74]. The epidermal equivalents graft is as effective as split-skin autograft in treatment of skin defects [75]. mAM loaded with HFSCs acts as another skin substitution [68]. This artificial skin could turn into a skin-like structure with well-survived cells when transplanted in a nude mice full-thickness skin defect model. The wound healed almost completely with a slight shrink after three weeks [68]. The Dermal papilla-like structures appeared in four weeks and become similar to normal skin tissue 6-8 weeks post-transplantation [68].

ESCs are critical in the substitution of hair follicles. Epidermal cells from embryonic mouse skin, mixed with follicular dermal cells, developed into hair-like structures *ex vivo*, which contained dermal papillae, hair matrix and rudimentary hair shafts. These structures developed further into mature hair follicles with a capacity of prolonged growth after implantation into mouse skin [76].

In contrast to the treatment of acute wounds, treatment of chronic ulcers has been much less effective. It was reported that treatment of chronic ulcer with epidermal equivalents generated from outer root sheath cells was successful with complete closure of the ulcers in about 45% of cases and a significant size reduction in other 40% within eight weeks [77]. Compared with stem cells derived from umbilical blood or bone marrow, epidermal stem cells possess an intrinsic privilege, for their strong capacity of giving rise to epidermis. Thus, ESC will be a more promising resource to treat chronic ulcer, diabetic foot and other complex skin defects.

## 6. ESCs in Peripheral Nerve Injury and Spinal Cord Injury

Among all the components of ESCs, only fPSCs can differentiate into neurons and glial cells [11–13,78]. After the nestin+ fPSCs were transplanted into nude mice subcutis or brain, some βIII-tubulin-positive-neuron progenies could survive for a long time [9,10]. Therefore, fPSCs exhibit a good potential for repair of peripheral nerve injury and spinal cord injury.

### 6.1. ESCs in Peripheral Nerve Injury

The clinical outcome for peripheral nerve injury is not yet satisfied. Peripheral nerve injury often results in long-term functional deficits, especially for those with nerve defects. Improving treatment outcomes remains an important clinical goal.

FPSCs exhibited strong potential in a mouse model of nerve injury [12,13,69]. Nestin+ fPSCs had been isolated from nestin-driven-GFP (ND-GFP) transgenic C57BL/6 mice hair follicle bulge, and then cultured and grown for 12 weeks. These cells were then injected directly into the repaired site of the severed sciatic nerve in C57BL/6 mice. Two months later, these fPSCs differentiated into GFAP+ Schwann cells, and formed a myelin sheath surrounding axons [79]. The gastrocnemius muscle contractions were recorded upon electrical stimulation. In the 6–12 weeks post-operation, the walking tracks exhibited significant improvement comparable to normal walking [69]. In another study, human nestin+ fPSCs were grown in suspending culture model for two months, and then were injected into the impinged fragment of the sciatic nerve in nude mice [28]. Eight weeks post-operation, these fPSCs differentiated into GFAP+ Schwann cells, which contained human DNA and human Ki-67 in the fragments of the injured sciatic nerve. These Schwann cells formed myelin sheaths surrounding axons, and the gastrocnemius muscle contractions were recorded upon electrical stimulation [28]. These studies demonstrated the following benefits of fPSCs after peripheral nerve injury: (1) both mouse and human fPSCs improve peripheral nerve regeneration in severed or impinged nerve; (2) undifferentiating fPSCs developed into functional Schwann cells post-transplantation; (3) fPSCs are able to improve mouse gait post-operation.

To treat a large defect of peripheral nerve, a nerve conduit with progenies of rat fPSCs was prepared. 500 ng/mL Sonic hedgehog (Shh) and 2000 nM retinoic acid were used to induce fPSCs into neurons, and 125 ng/mL neuregulin for induction into Schwann cells. The induction lasted seven days. FPSCs-derived GFP labeled neurons and Schwann cells, mixed at 1:1 ratio, were injected into the acellular nerve xenografts (ANXs), which were prepared from beagle sciatic nerve. Then ANXs were transplanted to bridge a 4 cm nerve defect in rat sciatic nerve. These III-tubulin+ neurons and s-100+ Schwann cells generated from fPSCs survived for 52 weeks post-operation. A significant improvement on myelinated fibers per square millimeter, the average thickness of myelin sheath, and the ratio of myelinated fibers in total nerve fibers appeared in ANX + cells group 52 weeks later, compared with that in ANX only group [79]. When these ANX with cells cultured for eight weeks, most of the surviving cells were S-100+ Schwann cells and some were Fuji+ neurons. These nerve conduits presented some electrophysiological features such as evoked potentials *in vitro* [80].

These animal experiments exhibit that fPSCs are able to improve nerve regeneration in peripheral nerve injury and nerve defect. These studies exhibited: (1) the progenies of fPSCs survived in ANX both *in vivo* and *ex vivo*; (2) the neurons and Schwann cells from fPSCs improved the nerve regeneration in ANX; (3) fPSCs have potential in the treatment of peripheral nerve defects. However, the authors did not report the changes on the gastrocnemius wet weight and the walking track of the rats. Currently there are no further studies on the mechanism or clinic application. Moreover, these fPSCs were cultured for a long time before transplantation, which hinders the potential application in clinic.

### 6.2. ESCs in Spinal Cord Injury

Spinal cord injury is a major challenge in orthopedic surgery. Limited treatment outcomes and serious dysfunction may keep patients bound to a wheelchair for the rest of life [81]. Current strategies in treating spinal cord injury include several modalities such as (1) tissue or cell transplantation; (2) providing growth-stimulating factors; (3) blocking the factors which inhibit neural regeneration and (4) modulation of inflammatory response following spinal cord injury [81].

Nestin+ fPSCs from ND-GFP transgenic C57B/6 mice grew in serum-free medium for two months, and were subsequently injected into the injured site at 10th thoracic spinal vertebrate of trans-severed spinal cord C57B/6 mice. Two month later, most of the GFP+ cells were GFAP+ and CNPase+ Schwann cells. These Schwann cells formed myelin sheaths surrounding the nerve axons. Twelve weeks post-operation, the mice recovered significantly better hind-limb function in locomotor rating scale, compared to the control mice without cell transplantation [29]. In another study, fPSCs were prepared from ND-GFP transgenic C57B/6 mice. A contused spinal cord C57B/6 mouse model was prepared at the midline of thoracic level 8 (T8) with 12 centi-Newtons (cN) for 3 s. Four months after fPSCs injection into left side of the contused spinal cord, there were a 24% improvement in sensory connectivity and a substantial recovery of touch perception via two examinations of spinal somatosensory evoked potentials (SpSEP) and the Semmes-Weinstein touch test [82]. Notably, bilateral functional improvements appeared after unilateral transplantation without fPSCs migration. Most of the transplanted cells differentiated into myelinating oligodendrocytes, and some developed a neuronal phenotype, but no Schwann cells or astrocytes. All progenies appeared near the injection site and some survived up to six months. Grafted cells integrated into the spinal tissue, intermingled with host neuritis in the injured spinal cord, and enhanced scar vascularization. Bilateral functional improvements suggested that EPI-NCSCs could exert their function by secreting diffusible molecules, including neurotrophins, angiogenic factors and metalloproteases [65,82,83].

These studies reveal fPSCs can (1) survive and differentiate post-transplantation in trans-severed or contused spinal cord; (2) significantly improve the outcome after treatment; (3) exert their function through cell replacement, neuro-protection, angiogenesis and modulation of scar formation. While the time for preparing grafted cells is quite long, there is currently no clinic application report on fPSCs.

A comparative study was designed to compare the pluripotent stem cells from hair follicle bulge (fPSCs) with those from dermal papilla, on their role of repairing the trans-severed spinal cord. The result demonstrated that both types of pluripotent stem cells differentiated into neuronal and glial cells, attached to the surrounding tissue very well, and constructed the connection with host tissue. Both groups significantly improved locomotor function recovery, with the bulge area being the greater and more constant source [84].

Without any induction, nestin+ fPSCs can express about 18 molecular markers of immature neural crest cells, including *Oct4*, *Nanog*, *Sox2*, *et al.* [8–10]. Most fPSCs differentiate into neural cells after transplanted into spinal cord [65,83]. After transplanted into the subcutis that is non-neural tissue in nude mice, fPSCs could differentiate into βIII-tubulin+ neurons [10]. This suggests fPSCs have a strong capacity for differentiating into neural cells compared with other adult stem cells.

## 7. ESCs in Bone and Muscle

Several studies reported that fPSCs could differentiate into smooth muscle cells, muscle, chondrocytes, and osteocyte lineages *in vitro* [9,24,85]. Human fPSCs expressed some osteogenic early lineage markers in nature, such as osteocalcin, osteopontin and collagen type 1 at a low level. Cultured in osteogenic medium, human fPSCs significantly increased the level of the expression with osteocalcin, osteopontin, collagen type 1, and maintain at a high level. Alizarin Red S staining, an indicator of calcium deposits was intensely positive in cultures [85]. Currently, there are no reports on fPSCs osteogenesis *in vivo*, comparing to many pre-clinical and clinical reports with the bone marrow stem cells. Only one study reported the application of fPSCs in muscle. After being transplanted into skeletal muscle, fPSCs survived in the muscle for 4 months, some fused with muscle fiber, while others became neuron-like cells within the muscle [27]. Although not as promising as bone marrow stem cells, ESCs still have a potential role in the treatment of bone and muscle disease.

### Epidermal Cell-Derived Induced Pluripotent Stem Cells (iPSCs)

Although not part of ESCs, iPSCs represent another important and effective approach in regenerative medicine to redirect somatic cells into another lineage via expression of lineage-specific genes through reprogramming. Epidermal cell is a good candidate to derive iPSCs because of easy accessibility [86–88], and is easier and more efficient compared to fibroblasts [89,90]. These iPSCs are able to differentiate into mature βIII-tubulin+TH+ dopaminergic neurons, βIII-tubulin+/TBR1+ forebrain glutamatergic neurons [91], and other neural lineages [92]. The functionally pluripotent neural crest (NC) stem-like cells from mature melanocytes expressed NC-related gene, and differentiated into multiple progenies, including osteoblasts, chondrocytes, smooth muscle cells, neurons, glial cells, and melanocytes [92]. iPSCs currently face challenges with regards to biological safety including oncogenesis [93]. Single transcription factor reprogramming is considered to be the possible solution.

## 8. Conclusions

ESCs have a strong capacity for giving rise to epidermis and differentiating into neural cells, and thus have strong potential as a therapeutic intervention in orthopedic regenerative medicine. HFSCs are able to differentiate into keratinocytes and form new epidermis after skin injury. fPSCs are able to differentiate into neurons, glial cells, smooth muscle cells, chondrocytes and osteocytes besides keratinocytes. ESCs play a critical role after cell replacement, or via artificial skin and nerve conduit. Therefore, ESCs have a profound potential in the treatment of orthopedic injuries and diseases, including the treatment of wounds, peripheral nerve injuries and spinal cord injuries.
